# Chitosan grafted methoxy poly(ethylene glycol)-poly(ε-caprolactone) nanosuspension for ocular delivery of hydrophobic diclofenac

**DOI:** 10.1038/srep11337

**Published:** 2015-06-12

**Authors:** Shuai Shi, Zhaoliang Zhang, Zichao Luo, Jing Yu, Renlong Liang, Xingyi Li, Hao Chen

**Affiliations:** 1 Institute of Biomedical Engineering, School of Ophthalmology & Optometry and Eye hospital, Wenzhou Medical University, 270 Xueyuan Road, Wenzhou 325027, China

## Abstract

This study aimed to develop a cationic nanosuspension of chitosan (CS) and methoxy poly(ethylene glycol)-poly(ε-caprolactone) (MPEG-PCL) for ocular delivery of diclofenac (DIC). MPEG-PCL-CS block polymer was synthesized by covalent coupling of MPEG-PCL with CS. The critical micelle concentration of the MPEG-PCL-CS block polymer was 0.000692 g/L. DIC/MPEG-PCL-CS nanosuspension (mean particle size = 105 nm, zeta potential = 8 mV) was prepared and characterized by Fourier transform infrared spectroscopy, X-ray diffraction, and differential scanning calorimetry. The nanosuspension was very stable without apparent physical property changes after storage at 4 °C or 25 °C for 20 days, but it was unstable in the aqueous humor solution after 24 h incubation. Sustained release of the encapsulated DIC from the nanosuspension occurred over 8 h. Neither a blank MPEG-PCL-CS nanosuspension nor a 0.1% (mass fraction) DIC/MPEG-PCL-CS nanosuspension caused ocular irritation after 24 h of instillation. Enhanced penetration and retention in corneal tissue was achieved with a Nile red/MPEG-PCL-CS nanosuspension compared with a Nile red aqueous solution. *In vivo* pharmacokinetics studies showed enhanced pre-corneal retention and penetration of the DIC/MPEG-PCL-CS nanosuspension, which resulted in a higher concentration of DIC (C_max_) in the aqueous humor and better bioavailability compared with commercial DIC eye drops (*P* < 0.01).

Currently, topical administration is the first choice for treatment of ocular diseases because of its convenience and patient compliance^1^,^2^,^3^,^4^. However, clinically approved formulations (e.g., eye drops, ointments) suffer from low bioavailability after topical administration because of the anatomical and physiological barriers of eye that prevent entry of foreign compounds. These mechanisms include rapid drainage and tear turnover, and can decrease the total quantity of drug reaching the intraocular tissue by 5%. To overcome this, high-dosage administration frequently occurs, and this might be associated with a higher risk of side effects and lower patient compliance^5^,^6^,^7^. Therefore, the development of an efficient topical formulation with the capability to deliver the drug at the correct dosage without frequent administration is an important challenge for pharmaceutical scientists.

Over the past three decades, considerable attention has been focused on the development of an efficient topical formulation to improve the bioavailability of ophthalmic drugs^8^,^9^,^10^,^11^. One strategy has been to prolong the residence time of the drug in contact with the corneal tissue by using biodegradable and biocompatible polymeric hydrogels^12^,^13^,^14^. In recent years, researchers have successfully developed various formulations based on *in situ* gelling systems, such as thermosensitive and pH sensitive hydrogels, that can increase the ocular residence time through their enhanced viscosity and mucoadhensive properties and thus improve the bioavailability of the ophthalmic drug^10^,^11^,^15^. More recently, two commercial products, Timoptic-XE^®^ and Zirgan^®^, based on *in-situ* gelling have been launched in the US for treatment of ocular diseases^16^. Timoptic-XE^®^ is an ion-activated gelling system that encapsulates timolol for the treatment of glaucoma. Zirgan^®^ is a pH sensitive hydrogel loaded with ganciclovir for herpetic keratitis therapy.

Another strategy to improve the ocular bioavailability of drugs after topical administration is to use nanomedicine and nanotechnology that allow the drug molecules to intimately interact with specific ocular tissues, to overcome the corneal barrier, and to increase the penetration of drugs across corneal tissue^5^,^6^,^7^,^8^,^17^,^18^. Over the past two decades, nanocarriers, including polymeric nanoparticles/nanospheres, lipid-based micro/nano emulsions, liposomes and dendrimers, have been designed and fabricated for ocular drug delivery applications. Among these nanocarriers, nanoparticles/nanospheres formed from biodegradable polymers, such as polycaprolactone, poly(lactic-co-glycolic acid), poly(ethyleneglycol)-poly(caprolactone), have attracted considerable attention because they are easy to prepare and the preparation methods can be scaled up^7^,^19^,^20^,^21^. Pilot studies suggest that the particle size and surface properties greatly affect the ability of nanoparticles to interact with the ocular mucosa. Generally, particles smaller than 10 μm exhibit reduced eye irritation, reduced tearing, and enhanced corneal penetration compared with larger particles (>10 μm), yet result in the better drug bioavailability^22^. Meanwhile, positively charged nanoparticles show prolonged retention times on the corneal surface compared with negatively charged nanoparticles because of possible electrostatic interactions of the nanoparticles with negatively charged mucin^1^,^2^. Indeed, our previous studies demonstrated that the corneal penetration of diclofenac (DIC) encapsulated in methoxy poly(ethyleneglycol)-poly(caprolactone) (MPEG-PCL) nanoparticles (particle size 50 nm) was 17-fold higher than that of a DIC phosphate buffered saline solution^7^. However, rapid clearance of DIC/MPEG-PCL nanoparticles was mainly attributed to the nearly neutral charge of the nanoparticles. It is well known that chitosan (CS) as a cationic polysaccharide exhibits favorable properties, such as mucoadhesion and permeability, that have led to its widespread use in the biomedical field.

The aim of this work was to develop a novel block polymer composed of cationic CS and MPEG-PCL, which could self-assemble into cationic micelles for payload of hydrophobic drugs. Possible electrostatic interactions of the micelles with the negatively charged mucin should increase the pre-corneal retention and improve the bioavailability of the drug.

## Results and Discussion

### Synthesis and characterization of MPEG-PCL-CS block polymer

**Figure 1:**
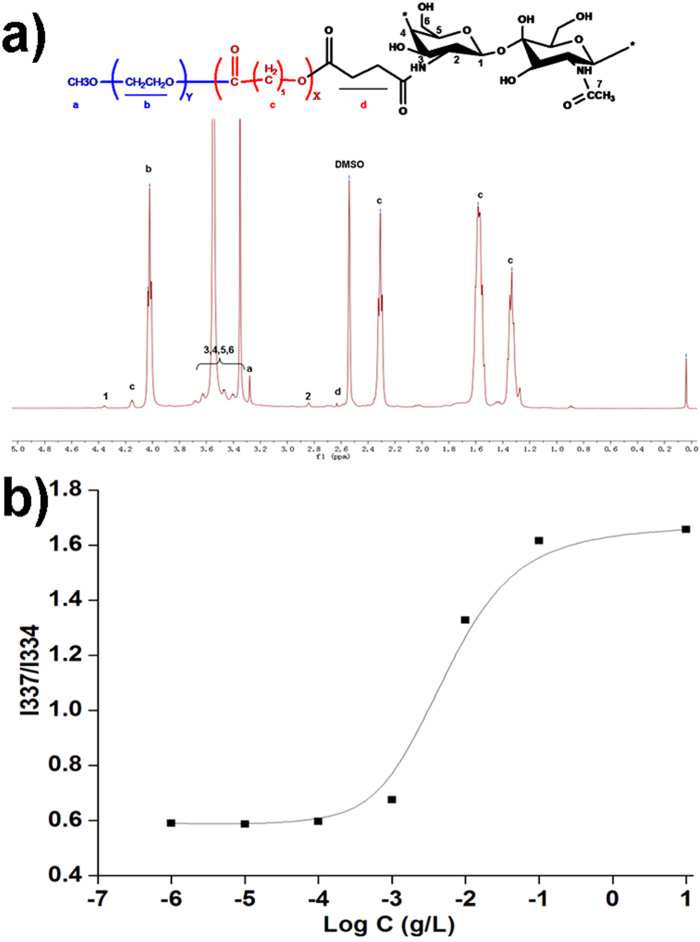
**a**) ^1^H-NMR spectrum of the MPEG-PCL-CS block polymer; **b**) intensity ratio plots of I_337_/I_334_ vs. log C for MPEG-PCL-CS.

Hydrogen nuclear magnetic resonance spectroscopy (^1^H-NMR) was used to confirm successful synthesis of the MPEG-PCL-CS block polymer (Fig. 1a). The ^1^H-NMR spectrum of the MPEG-PCL-CS block polymer showed characteristic signals for MPEG-PCL (1.33, 1.58, 2.31, 3.28, 4.03 and 4.15 ppm) and CS (4.36 ppm, and broad peaks between 3.31 and 3.72 ppm). A weak signal was observed at 2.62 ppm for protons from succinate, which was used to link the MPEG-PCL and CS. The ^1^H-NMR results indicated the synthesis of the MPEG-PCL-CS block polymer was successful.

Because the MPEG-PCL-CS block polymer was amphiphilic, it could self-assemble into a nanosuspension spontaneously in an aqueous solution. The critical micellar concentration (CMC) of MPEG-PCL-CS was investigated by the fluorescence probe method^23^. The change in the total fluorescence intensity (I) was negligible change at low MPEG-PCL-CS concentrations. At higher MPEG-PCL-CS concentrations, the total fluorescence intensity (I) increased, which indicated pyrene was incorporated into the core of the nanoparticles. The I_337_/I_334_ intensity ratio was used to calculate the CMC of MPEG-PCL-CS in aqueous solution (Fig. 1b). The CMC of MPEG-PCL-CS (0.000692 g/L) was lower than for other low molecular weight surfactants, including for sodium dodecyl sulfonate (2 g/L) and tetradecyl trimethyl ammonium bromide (1.3 g/L), but higher than for other amphiphilic polymers, such as MPEG-PCL (10^−7^ g/L)^24^.

### Preparation and characterization of the DIC/MPEG-PCL-CS nanosuspension

**Figure 2:**
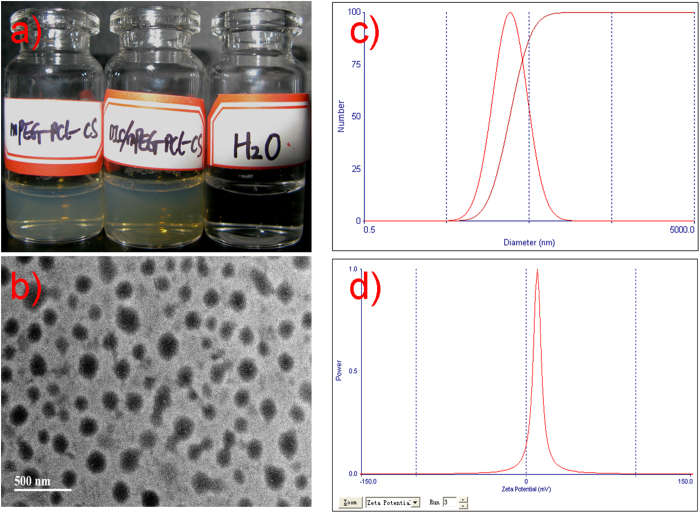
**a**) Representative images of a blank MPEG-PCL-CS nanosuspension and a DIC/MPEG-PCL-CS nanosuspension; **b**) TEM image of a DIC/MPEG-PCL-CS nanosuspension; **c**) size distribution of the DIC/MPEG-PCL-CS nanosuspension; d) Zeta potential of the DIC/MPEG-PCL-CS nanosuspension.

The self-assembling capability of the MPEG-PCL-CS block polymer (CMC <10^−5^ g/L) could be used to encapsulate a hydrophobic drug such as DIC. Encapsulation of DIC would form a DIC/MPEG-PCL-CS nanosuspension. Both the MPEG-PCL-CS nanosuspension and DIC/MPEG-PCL-CS nanosuspension were transparent with a slight opalescence. Loading hydrophobic DIC into the MPEG-PCL-CS nanosuspension did not change its color. A transmission electron microscope (TEM) image (Fig. 2b) indicated that the DIC/MPEG-PCL-CS nanosuspension was composed of uniform spheres with diameters of 100–150 nm. Dynamic light scattering showed the mean particle size of the DIC/MPEG-PCL-CS nanosuspension was 105 nm and the zeta potential was +8 mv (Fig. 2c,d). The weak positive charge of the DIC/MPEG-PCL-CS nanosuspension could be attributed to the presence of cationic CS on the surface of nanoparticles, which could facilitate effective adhesion to the corneal surface and prolong the residence time of the formulation^25^. Generally, the normal osmotic pressure of plasma is around 330 mOsm/L. In the present study, the osmotic pressure of the DIC/MPEG-PCL-CS nanosuspension in aqueous solution and the DIC/MPEG-PCL-CS nanosuspension in a 0.9% NaCl solution were 1 ± 0.3 mOsm/L and 296 ± 3 mOsm/L, respectively (Table 1). With the 0.9% NaCl solution, the DIC/MPEG-PCL-CS nanosuspension was isotonic with plasma, and therefore could be used in practical applications. The viscosity values of DIC commercial eye drops, DIC/MPEG-PCL-CS nanosuspension in aqueous solution, and DIC/MPEG-PCL-CS nanosuspension in 0.9% NaCl solution and were 0.023 ± 0.008 Pa·s, 0.018 ± 0.006 Pa·s and 0.021 ± 0.007 Pa·s, respectively. This result indicated that the DIC/MPEG-PCL-CS nanosuspension had similar viscosity with that of DIC commercial eye drops. With a DIC to MPEG-PCL-CS polymer mass ratio of 5 to 95, the loading capacity (LC) of formulation was 4.8% and the loading efficiency (LE) reached 98.5%.

### FTIR analysis

**Figure 3:**
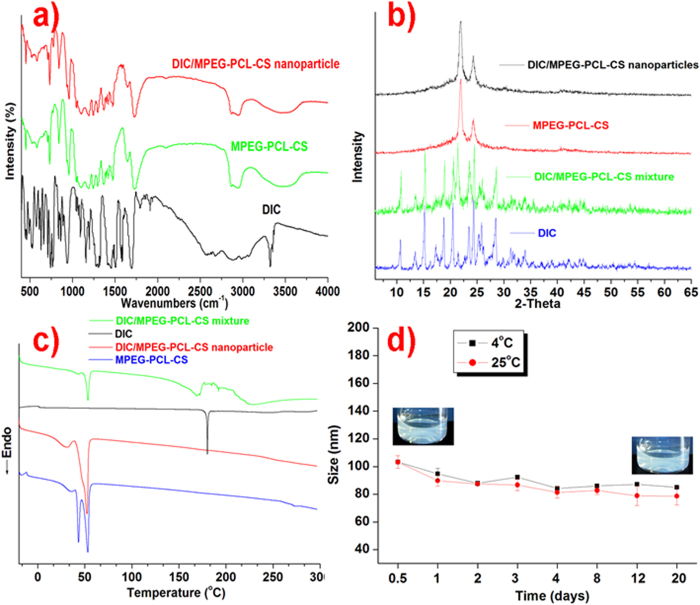
a) FTIR spectra of DIC, MPEG-PCL-CS and DIC/MPEG-PCL-CS nanoparticles; **b**) XRD pattern of DIC, MPEG-PCL-CS, DIC/MPEG-PCL-CS mixture and DIC/MPEG-PCL-CS nanosuspension; **c**) DSC profiles of DIC, MPEG-PCL-CS, DIC/MPEG-PCL-CS mixture and DIC/MPEG-PCL-CS nanosuspension; **d**) stability of the DIC/MPEG-PCL-CS nanosuspension at 4 °C and 25 °C.

Possible interactions between the drug and the polymer carrier were investigated by FTIR analysis (Fig. 3a). In the spectrum of DIC, characteristic peaks were observed at 1450–1600 cm^−1^ for benzene C=C stretching, 3340 cm^−1^ for N-H stretching, and 1790 cm^−1^ for –C=O stretching. The FTIR spectrum of the MPEG-PCL-CS block polymer showed peaks at 1723 cm^−1^ for –C=O stretching and 2800–3000 cm^−1^ for C-H stretching. When comparing the FTIR spectra of DIC and the MPEG-PCL-CS block polymer with the spectrum of the DIC/MPEG-PCL-CS nanoparticles, no distinctive peaks changes were observed. This suggested that there was no intermolecular reaction between DIC and the MPEG-PCL-CS in the nanosuspension.

### X-ray diffraction (XRD) analysis

The XRD pattern (Fig. 3b) for DIC showed characteristic peaks at 2θ 10.6°, 15.2°, 18.8°, 20.6°, 24.5° and 28.5°, and that of the blank MPEG-PCL-CS block polymer showed peaks at 2θ 21.3° and 23.5°. The XRD spectrum of a DIC/MPEG-PCL-CS mixture exhibited peaks for both DIC and the MPEG-PCL-CS block polymer. This result indicated that physical blending did not affect the drug diffraction. By comparison, the pattern of the DIC/MPEG-PCL-CS nanosuspension showed suppression of the DIC peaks, which suggests the drug crystallinity decreased in the nanosuspension.

### Differential scanning calorimetry (DSC)

DSC is one of the most effective approaches to study physical interactions between drugs and the matrix in a formulation. The DSC profiles are presented in Fig. 3c. For DIC, a single endothermic peak at 182 °C was observed, while for the blank MPEG-PCL-CS block polymer endothermic peaks were observed at 43 °C and 52 °C. DSC of the DIC/MPEG-PCL-CS mixture showed endothermic peaks for both DIC and MPEG-PCL-CS. A slight shift in the position of the endothermic peak of DIC (182 °C to 172 °C) was observed for the DIC/MPEG-PCL-CS mixture, and this could be attributed to solubilization of DIC into matrix and solid state interaction induced by the heating process^7^,^20^. By contrast, no DIC was detected in the DSC profile of the DIC/MPEG-PCL-CS nanosuspension, which indicated that the drug crystallinity decreased in the formulation.

### Stability test

Particle size distribution is an important parameter for evaluating stability, and it plays an important role in the ability of a drug to cross the corneal barrier. The mean diameter of the DIC/MPEG-PCL-CS nanosuspension did not change over 20 days of storage at 4 °C or 25 °C (Fig. 3d). In addition, no obvious precipitates were observed in the DIC/MPEG-PCL-CS nanosuspension with storage for up to 20 days. These results indicate that the hydrophobic DIC incorporated into the hydrophobic core of the MPEG-PCL-CS is very stable. The particle size of the nanosuspension increased to around 200 nm after 24 h incubation. This change might be induced by electrostatic interaction between the cationic nanosuspension and anionic protein in the aqueous humor.

### *In vitro* cytotoxicity test

**Figure 4:**
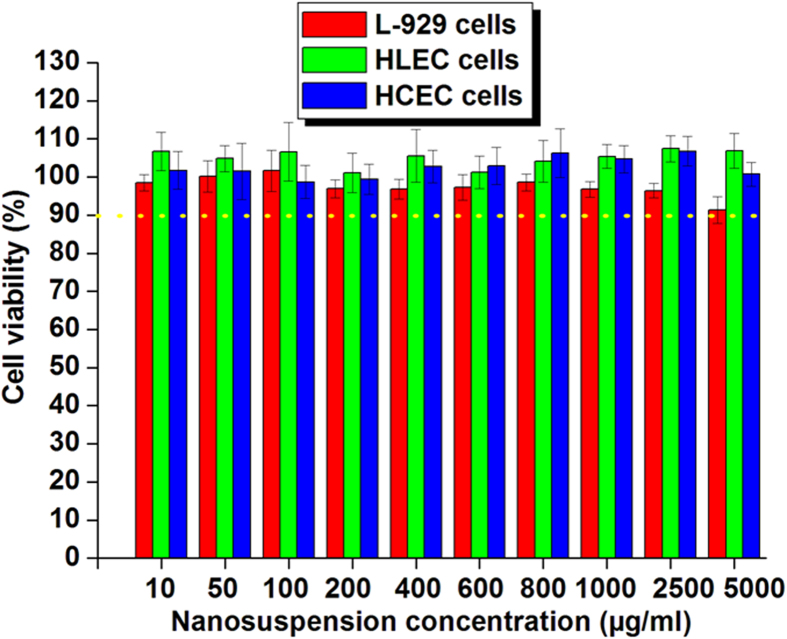
*In vitro* cytotoxicity of the MPEG-PCL-CS nanosuspension against various cell lines (L-929 cells, HLEC cells and HCEC cells) after 24-h incubation.

A versatile nanocarrier for ocular drug delivery must be capable of delivering drugs to the target tissue without compromising the viability of the host cells. Pilot studies have indicated that cationic nanoparticles could disrupt the cell membrane and cause cytotoxic effects^8^,^26^. Therefore, it was very important to investigate the effect of the developed cationic MPEG-PCL-CS nanosuspension on the cell viability of human corneal epithelial cells (HCEC), human lens epithelial cells (HLEC), and L-929 cells. The MPEG-PCL-CS nanosuspension had no apparent cytotoxicity against HCEC, HLEC and L-929 cells (cell viability >90%) after 24-h incubation with MPEG-PCL-CS nanosuspension concentrations up to 5 mg/mL (Fig. 4). Therefore, the cationic MPEG-PCL-CS nanosuspension could be considered as a non-toxic nanocarrier for ophthalmic drug delivery.

### *In vitro* release study

**Figure 5:**
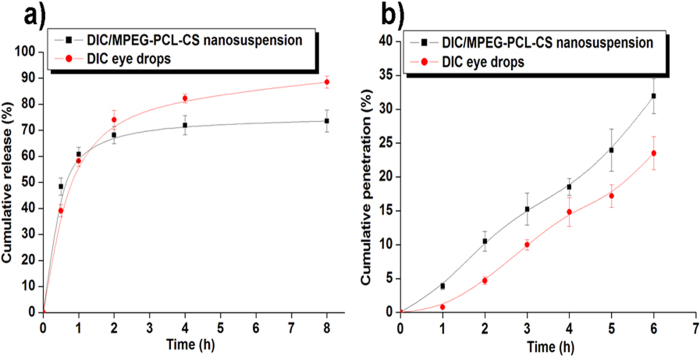
a ) *In vitro* release behavior of DIC from 0.1% (mass fraction) DIC commercial eye drops and 0.1% (mass fraction) DIC/MPEG-PCL-CS nanosuspension in PBS (pH 7.4) at 37 °C; **b**) *In vitro* corneal penetration profiles of 0.1% (mass fraction) DIC commercial eye drops and 0.1% (mass fraction) DIC/MPEG-PCL-CS nanosuspension.

Figure 5a depicts the *in vitro* release profile of DIC from DIC commercial eye drops and the DIC/MPEG-PCL-CS nanosuspension. The release profile of DIC from the DIC/MPEG-PCL-CS nanosuspension was similar to that from the eye drops. Both these two formulations showed a two-stage release, with an initial rapid release followed by a slower sustained release. The initial rapid release of DIC from the DIC commercial eye drops and DIC/MPEG-PCL-CS nanosuspension resulted in release of 39% and 47% of the total DIC content in the first 0.5 h, respectively. From a clinical point of view, an initial rapid release in the early stages is beneficial for the therapeutic effect because it helps achieve a therapeutic concentration of the drug in minimal time, and this is followed by sustained release of drug to maintain a minimal effective concentration^22^,^27^. With the sustained release, 73% of the total DIC was released from the MPEG-PCL-CS nanosuspension at 8 h, while 88% of the total DIC was released from the commercial DIC eye drops at 8 h. It is well known that drug release from polymeric nanoparticles is a complicated process that is affected by several factors, including drug diffusion, nanocarrier degradation, and affinity between the drug and nanocarrier^1^,^7^,^20^,^21^,^22^. Interestingly, the DIC commercial eye drops also exhibited a sustained release stage, which could be attributed to excipients such as carboxymethylcellulose in the eye drops that could contribute to hydrogen bond formation and ionic interactions.

### *In vitro* corneal penetration test

Figure 5b illustrates the *in vitro* corneal penetration results for DIC commercial eye drops and the DIC/MPEG-PCL-CS nanosuspension. The cumulative penetration of DIC from both DIC formulations exhibited a nearly liner relationship with time, indicating that passive diffusion was the main penetration mechanism of DIC through the cornea, which was in accordance with previous studies. previous studies^8^,^22^,^28^. The developed DIC/MPEG-PCL-CS nanosuspension showed enhanced penetration of DIC compared with the DIC commercial eye drops. Specifically, after 6 h of *in vitro* penetration, the cumulative penetration of DIC for the commercial DIC eye drops and DIC/MPEG-PCL-CS nanosuspension were 23% and 32%, respectively. The penetration with the DIC/MPEG-PCL-CS nanosuspension was approximately 1.4 times higher than that with the commercial DIC eye drops. It has been reported that amphiphilic nanoparticles with diameters less than 10 μm could distribute throughout the cornea easily, and in aid distribution of incorporated drugs^2^,^5^,^7^. The particles in the developed DIC/MPEG-PCL-CS nanosuspension were about 100 nm in diameter, which might be able to explain the enhanced penetration of DIC from the nanosuspension. Furthermore, the weak positive charge of the DIC/MPEG-PCL-CS nanosuspension (8 mV) might result in interaction with negatively charged mucin on the corneal surface, which could further enhance the corneal retention of DIC *in vivo* compared with DIC commercial eye drops^16^,^17^,^20^. Therefore, it is reasonable to believe that the developed DIC/MPEG-PCL-CS nanosuspension could increase the bioavailability of DIC *in vivo* after topical ocular administration.

### Ocular irritation test

The results of an irritation test are presented in Table 2. After 1 h of instillation of various formulations, the 0.1% (mass fraction) DIC commercial eye drops, blank MPEG-PCL-CS nanosuspension, and 0.1% (mass fraction) DIC/MPEG-PCL-CS nanosuspension showed a certain level of irritation. This was followed by recovery to normal after 6 h. The temporary irritation caused by the MPEG-PCL-CS nanosuspension might be induced by its cationic properties. No other ocular damage or clinically abnormal signs were observed in the cornea, conjunctiva or iris after the 24-h instillation, which suggests that the ocular irritation was temporary (**Fig. S-1**).

### *In vivo* corneal penetration test

**Figure 6:**
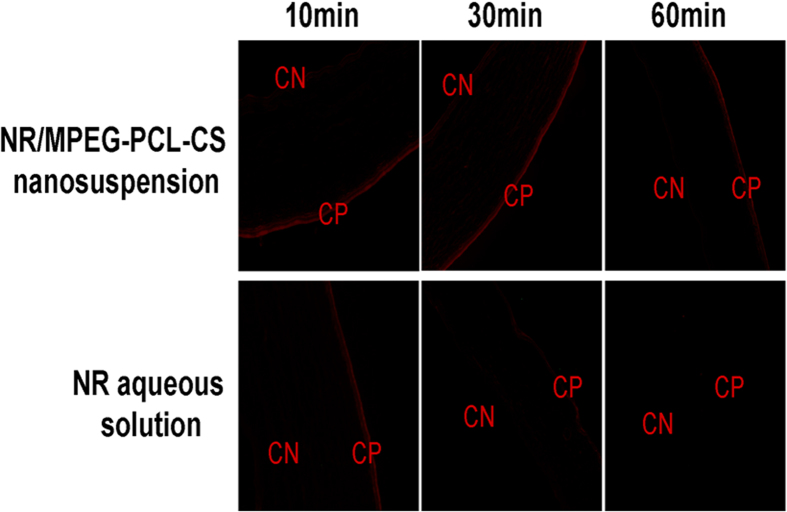
Fluorescence microscopy of rabbit cornea after treatment with Nile Red (NR) aqueous solution (10, 30 and 60 min) and a NR/MPEG-PCL-CS nanosuspension (10, 30 and 60 min). CN = corneal endothelium layer, and CP = corneal epithelium layer.

To assess the capability of various formulations to enhance corneal penetration, fluorescence in the corneal tissue after instillation of a Nile red (NR) aqueous solution and NR/MPEG-PCL-CS nanosuspension were observed by an inverted fluorescence microscope (Fig. 6). In the 10 min after instillation of the NR aqueous solution or NR/MPEG-PCL-CS nanosuspension, the fluorescence was mainly concentrated in the corneal epithelium (CP) and the fluorescence intensity was similar for both formulations. As the time after instillation increased, the fluorescence gradually moved to the corneal endothelial (CN) layer for the NR/MPEG-PCL-CS nanosuspension, while the fluorescence in the corneal tissue was attenuated for the NR aqueous solution. As expected, the NR/MPEG-PCL-CS nanosuspension showed higher fluorescence intensity than of the NR aqueous solution even 60 min after instillation, which suggests that MPEG-PCL-CS nanosuspension resulted in enhanced penetration and retention . Previous studies have demonstrated that CS or CS-modified nanocarriers could open the tight junction of corneal epithelial cells, and through this, enhance corneal penetration^28^,^29^,^30^. Furthermore, cationic CS in the nanosuspension could interact with negatively charged mucin on the corneal surfaces, which would enhance retention^18^,^31^.

### *In vivo* pharmacokinetics

**Figure 7:**
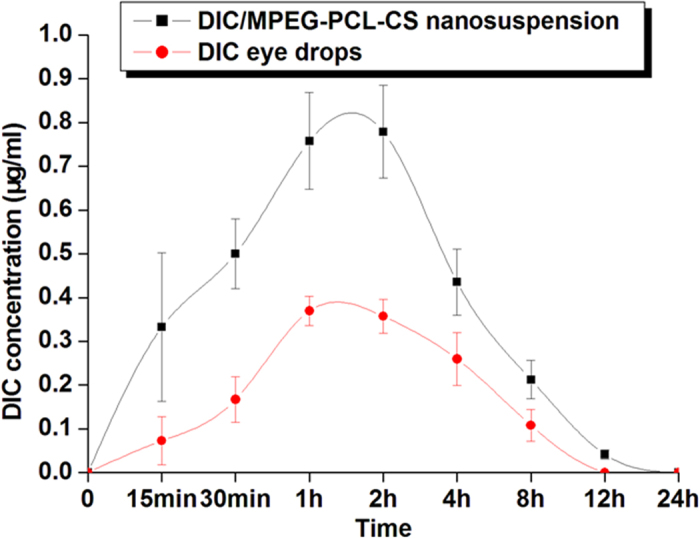
DIC concentration profiles in rabbit aqueous humor after instillation of 50 μL of either a 0.1% (mass fraction) DIC commercial eye drops or 0.1% (mass fraction) DIC/MPEG-PCL-CS nanosuspension.

The *in vivo* pharmacokinetics results for various DIC formulations are shown in Fig. 7. The DIC/MPEG-PCL-CS nanosuspension resulted in much higher concentration of DIC (C_max_) in the aqueous humor than commercial DIC eye drops (Table 3). The enhanced C_max_ for the DIC/MPEG-PCL-CS nanosuspension (0.78 ± 0.11 μg/mL) compared with the commercial DIC eye drops (0.37 ± 0.03 μg/mL) could be attributed to enhanced corneal penetration of the nanosuspension. The level of DIC in the aqueous humor was maintained for up to 12 h with the DIC/MPEG-PCL-CS nanosuspension because of its pre-corneal retention, as indicated by the *in vivo* corneal penetration test. The area under the aqueous humor concentration curve of the DIC/MPEG-PCL-CS nanosuspension was 2.3 times higher than that of the commercial DIC eye drops (*P* < 0.01). All these results suggested that the developed DIC/MPEG-PCL-CS nanosuspension could greatly improve drug bioavailability after topical administration.

## Methods

### Synthesis and characterization of the MPEG-PCL-CS block polymer

According to an established protocol^7^, MPEG-PCL block polymer was synthesized by ring-opening polymerization of ε-caprolactone at 130 °C for 24 h initiated by MPEG using stannous octoate as the catalyst. The obtained MPEG-PCL block polymer (15 g) were dissolved in 200 mL of tetrahydrofuran, followed by addition of 0.9 g of succinic anhydride and 0.9 g of 4-dimethylaminopyridine. This mixture was reacted at room temperature for 48 h under a stream of nitrogen gas. Finally, the reaction mixture was precipitated with excess cold petroleum ether and the precipitate was isolated and dried in a vacuum desiccator to obtain the MPEG-PCL-COOH block polymer powder.

To obtain MPEG-PCL-CS block polymer, MPEG-PCL-COOH was coupled with CS (food grade, Mw 2000) using DCC and NHS as coupling agents. CS and MPEG-PCL-COOH at a mass ratio of 1:20 were dissolved in very dry dimethylsulfoxide (DMSO). This was followed by addition of DCC (2 molar equivalents to MPEG-PCL-COOH) and NHS (1.2 molar equivalents to MPEG-PCL-COOH), and the mixture was reacted at room temperature for 48 h. This was followed by dialysis with DMSO for 3 days, and excess distilled water solution for 7 days. Finally, the powder product was obtained by lyophilization.

### H-NMR analysis

The ^1^H-NMR spectra of the samples were obtained with a Varian 400 spectrometer (Varian, Palo Alto, CA) at 400 MHz using DMSO-*d*_6_ as the solvent, and tetramethylsilane as the internal reference.

### CMC measurement

The CMC of the MPEG-PCL-CS block polymer was determined by fluorescence spectroscopy with pyrene as a hydrophobic fluorescence probe. A series of MPEG-PCL-CS nanosuspensions with concentrations ranging from 10^−6^ to 1 mg/mL were prepared as follows. First, 1 mL of a pyrene acetone solution was added to a 50 mL test tube, and then evaporated under a stream of nitrogen gas. An aliquot of the MPEG-PCL-CS nanosuspension (10 mL) with a concentration between 10^−6^ and 1 mg/mL was added to give a final pyrene concentration of 6 × 10^−7^ mol/L. These solutions were incubated at room temperature overnight. The fluorescence excitation spectra were measured at an emission wavelength of λ = 373 nm. The ratios of the fluorescence intensities at 337 and 334 nm (I_337_/I_334_) were used to determine the CMCs.

### Preparation of the DIC/MPEG-PCL-CS nanosuspension

The DIC/MPEG-PCL-CS nanosuspension was prepared by a co-incorporation method as reported previously^7^. First, 50 mg of DIC and 950 mg of the MPEG-PCL-CS block polymer were co-dissolved in 5 mL of an organic solvent (e.g. acetone, dichloromethane). This was followed by removal of the organic solvent from the system on a rotary evaporator. The resulting residue was re-suspended in 20 mL of distilled water in a water bath at 50 °C to form the DIC/MPEG-PCL-CS nanosuspension. Finally, the DIC/MPEG-PCL-CS nanosuspension was lyophilized to obtain the DIC/MPEG-PCL-CS powder for further study. The viscosity and osmotic pressure of various formulations (DIC commercial eye drops and the DIC/MPEG-PCL-CS nanosuspension) were measured using a AR-2000 rheometer (TA Instruments, New Castle, DE) in flow mode (shear rate 1 s^–1^) and a direct membrane osmometer (Cryobasic, Astori Tecnica s.n.c., Poncarale, Italy), respectively. The drug LC and LE were calculated by the following the equations:
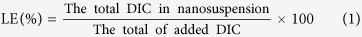

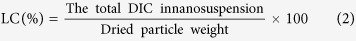


### Characterization

#### FTIR spectra

FTIR spectra were obtained using a spectrometer with KBr pellets from 400 to 4000 cm^–1^.

#### XRD spectra

XRD spectra were measured on an X-ray diffractometer (D8 Advance, Bruker AXS GmbH, Karlsruhe, Germany) using CuKα radiation. The scanning range was 5° to 65° and scanning rate was 5°/min.

#### DSC

DSC of DIC, the MPEG-PCL-CS block polymer, the DIC/MPEG-PCL-CS mixture, and the DIC/MPEG-PCL-CS nanosuspension powder were performed using a Q-2000 DSC (TA Instruments). The heating rate was 10 °C/min, and measurements were recorded from −20 °C to 300 °C.

#### Size distribution and zeta potential measurement

To determine the size distribution and zeta potential of the DIC/MPEG-PCL-CS nanosuspension, samples were diluted with distilled water and analyzed with a Zetasizer (ZetaPlus, Brookhaven Instruments Corporation, Holtsville, NY).

#### TEM observation

The morphology of the DIC/MPEG-PCL-CS nanosuspension was observed by TEM. The samples were placed on a copper grid and negatively stained with phosphotungstic acid (0.5% mass fraction) for TEM observations.

### Stability test

Briefly, a set mass of DIC/MPEG-PCL-CS was dissolved in a certain volume of distilled water to form a 0.1% (mass fraction) DIC/MPEG-PCL-CS nanosuspension. Then, 5 mL of the 0.1% (mass fraction) DIC/MPEG-PCL-CS nanosuspension was stored at 25 °C or 4 °C for 20 days for an *in vitro* stability study (*n* = 3). Color changes, turbidity and precipitation were carefully monitored over the storage period. Furthermore, an *in vivo* stability test of the 0.1% (mass fraction) DIC/MPEG-PCL-CS nanosuspension was performed by mixing it with an equal volume of aqueous humor. This mixture was incubated at 37 °C and changes in the particle size with time were monitored in a period of 3days.

### *In vitro* cytotoxicity test

The MTT assay was employed to evaluate the *in vitro* cytotoxicity of the MPEG-PCL-CS nanosuspension against HCEC, HLEC, and L-929 cells. Briefly, cells were seeded at a density of 1 × 10^4^ cells/well in 96-well plates and incubated for 24 h in a 5% CO_2_ incubator. Then, aliquots of the MPEG-PCL-CS nanosuspension ranging in concentration from 10 to 5000 μg/mL were added to the wells, followed by incubation for another 24 h. Untreated cells in growth medium were used as controls. After 24 h of incubation, 20 μL of MTT solution (5 mg/mL in phosphate buffered saline (PBS)) was added to each well, and the plates were incubated for an additional 2 h. The MTT solution was then carefully removed and DMSO was added. The absorbance values were recorded using an ELISA microplate reader (Bio-Rad, Hercules, CA) at 570 nm. Cell viability (%) was calculated according to the following equation: cell viability (%) = absorbance test/absorbance control × 100%, where the absorbance control was the absorbance for the control wells. All data are expressed as the mean of six measurements (mean ± SD, *n* = 6).

### *In vitro* release study

The *in vitro* release behavior of DIC from 0.1% (mass fraction) DIC commercial eye drops and the 0.1% (mass fraction) DIC/MPEG-PCL-CS nanosuspension was studied using a dialysis bag method. Briefly, 0.5 mL of 0.1% (mass fraction) DIC commercial eye drops or 0.1% (mass fraction) DIC/MPEG-PCL-CS nanosuspension was sealed in a dialysis bag (molecular weight cut-off 3500). The dialysis bag was immersed in 20 mL of PBS (pH 7.4) at 37 °C. At predetermined intervals, 3 mL aliquots of the release medium were withdrawn for quantitative determination of DIC by high-performance liquid chromatography (HPLC). The residual release medium was also removed at this time, and discarded. After each removal of release medium, 20 mL of fresh pre-warmed PBS was added to the immersion solution.

### *In vitro* corneal penetration test

*In vitro* corneal penetration tests were performed on freshly excised rabbit cornea. All these tests compiled with the Guide for the Care and Use of Laboratory Animals, Institute of Laboratory Animal Resources, and were approved by the Institutional Animal Care and Use Committee of Wenzhou Medical University (Wehnzhou, China). Each rabbit cornea was carefully removed and immediately mounted on a Franz Diffusion cells between the donor and receptor compartment. The receptor compartment was filled with 5 mL of fresh PBS (pH 7.4), and the donor compartment was filled with 0.2 mL of either 0.1% (mass fraction) DIC commercial eye drops or 0.1% (mass fraction) DIC/MPEG-PCL-CS nanosuspension. The whole system was kept at 37 °C for 6 h. At specific intervals, 0.2 mL of the release medium from the receptor compartment was collected for detection of DIC by HPLC, and an equal volume of freshly prepared PBS was added to the receptor compartment.

### Ocular irritation test

Ocular irritation caused by the blank MPEG-PCL-CS nanosuspension, 0.1% (mass fraction) DIC commercial eye drops and 0.1% (mass fraction) DIC/MPEG-PCL-CS nanosuspension was evaluated by a modified Draize test using a slit lamp. Six male albino rabbits, each weighing approximately 2.5 kg, were used in the experiment. Fifty microliters of each sample was instilled directly into the lower conjunctival sac of the rabbit’s eye. A saline solution (0.9% NaCl) was used as the control. Congestion, swelling, discharge, and redness of the conjunctiva were scored and recorded by an experienced doctor after 1, 6 and 24 h of instillation.

### *In vivo* corneal penetration test

The capability of MPEG-PCL-CS nanosuspension to penetrate the cornea was evaluated by a fluorescence test in the rabbit’s eye. A fluorescent NR/MPEG-PCL-CS nanosuspension was prepared by the same method as for the DIC/MPEG-PCL-CS nanosuspension. A NR aqueous solution was used as the reference. The rabbit’s eye was instilled of 50 μL either the NR/MPEG-PCL-CS nanosuspension or NR aqueous solution on the corneal surface. After 10 min, 30 min and 60 min of instillation, the rabbit was scarified and the cornea tissue was excised and washed with PBS. A corneal cryostat section was prepared and imaged with a inverted fluorescence microscope.

### *In vivo* pharmacokinetics test

Six male New Zealand albino rabbits, each weighing approximately 2.5 kg, were used for *in vivo* pharmacokinetics tests. Six rabbits were randomly divided two groups, with three rabbits in each group. A single dosage (50 μL) of either 0.1% (mass fraction) DIC commercial eye drops or 0.1% (mass fraction) DIC/MPEG-PCL-CS nanosuspension was instilled into the lower conjunctival sac of the eye. After 15 min, 30 min, 1 h, 2 h, 4 h, 8 h, 12 h and 24 h of instillation, a 20 μL sample of the aqueous humor was collected using an insulin syringe (29 G needle). To remove protein, this sample was mixed with 80 μL of HPLC mobile phase and centrifuged as reported previously^27^,^32^. Finally, the DIC in the aqueous humor was quantified by HPLC (1200 series, Agilent, Santa Clare, CA). A reversed phase C18 column (4.6 × 150 mm,5 μm, ZORBAX Eclipse XDB-C18, Agilent) was used for sample separation. The mobile phase was acetonitrile–0.1% (mass fraction) triethylamine/phosphate buffer (65/35, *v*/*v*). The detection wavelength was 276 nm and the mobile phase flow rate was 1.0 mL/min. The obtained data were analysis by Drug and Statistics software (DAS 2.0).

## Additional Information

**How to cite this article**: Shi, S. *et al.* Chitosan grafted methoxy poly(ethylene glycol)-poly(ε-caprolactone) nanosuspension for ocular delivery of hydrophobic diclofenac *Sci. Rep.* **5**, 11337; doi: 10.1038/srep11337 (2015).

## Figures and Tables

**Table 1:** **Properties of the various formulations (** ***n*** **=** **3).**

**Formulations**	**Viscosity (Pa·s)**	**Osmotic pressure ( mOsm·L-1)**
DIC commercial eye drops	0.023 ± 0.008	302 ± 2
DIC/MPEG-PCL-CS nanosuspension in aqueous solution	0.018 ± 0.006	1 ± 0.3
DIC/MPEG-PCL-CS nanosuspension in 0.9% NaCl solution	0.021 ± 0.007	296 ± 3

**Table 2:** **Ocular irritation caused by various formulations to rabbits’ eyes as evaluated by the Draize test.**

**Formulations**	**1** **h**	**6** **h**	**24** **h**
Saline solution	0	0	0
Blank MPEG-PCL-CS nanosuspension	1( Conjunctival redness)	0	0
DIC commercial eye drops	1( Conjunctival redness)	0	0
DIC/MPEG-PCL-CS nanosuspension	1( Conjunctival redness)	0	0

**Table 3:** **Pharmacokinetic parameters of DIC in rabbit aqueous humor after instillation of 50** **μL of either 0.1% (mass fraction) DIC commercial eye drops or 0.1% (mass fraction) DIC/MPEG-PCL-CS nanosuspension.**

**Pharmacokinetic parameters**	**DIC/MPEG-PCL-CS nanosuspension**	**DIC commercial eye drops**
AUC_0–24_ (μg/mL h)	3.06 ± 0.57*	1.33 ± 0.23
C_max_ (μg/mL)	0.78 ± 0.11*	0.37 ± 0.03
T_max_ (h)	2	1.5

^*^Data were analyzed for statistical significance by one-way analysis of variance. *P* < 0.05 was considered as significant. *P* < 0.01 versus commercial DIC eye drops.
